# The A component (SmhA) of a tripartite pore-forming toxin from *Serratia marcescens*: expression, purification and crystallographic analysis

**DOI:** 10.1107/S2053230X20013862

**Published:** 2020-11-25

**Authors:** Alicia M. Churchill-Angus, Svetlana E. Sedelnikova, Thomas H. B. Schofield, Patrick J. Baker

**Affiliations:** a Department of Molecular Biology and Biotechnology, The University of Sheffield, Western Bank, Sheffield S10 2TN, United Kingdom; b Astbury Centre for Structural Molecular Biology, University of Leeds, Leeds LS2 9JT, United Kingdom

**Keywords:** pore-forming toxin, crystallization, ClyA family, *Serratia*

## Abstract

Tripartite α-pore-forming toxins are constructed of three proteins (A, B and C) and are found in many bacterial pathogens. While structures of the B and C components from Gram-negative bacteria have been described, the structure of the A component of a Gram-negative α-pore-forming toxin has so far proved elusive. SmhA, the A component from the opportunistic human pathogen *Serratia marcescens*, has been cloned, overexpressed and purified. Crystals were grown of selenomethionine-derivatized protein and anomalous data were collected. Phases were calculated and an initial electron-density map was produced.

## Abstract 2

SmhA, the A component of the tripartite α-pore-forming toxin from the opportunistic human pathogen *Serratia marcescens*, has been cloned, overexpressed and purified. Crystals were grown of selenomethionine-derivatized protein, anomalous data were collected, phases were calculated and an initial electron-density map was produced.

## 1. Introduction   

Tripartite α-pore-forming toxins (α-PFTs) are members of the ClyA α-pore-forming toxin family (Fagerlund *et al.*, 2008 ▸; Wilson *et al.*, 2019 ▸); however, unlike ClyA, where the pore is formed from an oligomer of a single protomer, three proteins (A, B and C) are involved in active pore formation (Sastalla *et al.*, 2013 ▸; Lindbäck *et al.*, 2004 ▸; Wilson *et al.*, 2019 ▸; Beecher & Macmillan, 1991 ▸). In the ClyA family the active pore is formed when the soluble protein(s) undergo a large-scale conformational change to expose the membrane-binding regions, with protomers assembling into a hydrophilic-lined pore (Benke *et al.*, 2015 ▸; Roderer & Glockshuber, 2017 ▸). It is proposed that each protein of the tripartite α-PFT (A, B and C) fulfils a role in the active pore that is provided by different regions of the ClyA protomer (Wilson *et al.*, 2019 ▸). The C component makes the first attachment to the target cell, binding to a single leaflet of the membrane, and is equivalent in function to the β-tongue region of soluble ClyA. In ClyA the pore is completed by the N-terminal amphipathic helix of each protomer assembling to construct the membrane-spanning, hydrophilic-lined pore of the oligomer (Roderer & Glockshuber, 2017 ▸; Wallace *et al.*, 2000 ▸; Benke *et al.*, 2015 ▸). In the tripartite α-PFTs the A and B components are functionally equivalent to this region of ClyA. The B component acts as the pore-forming unit, using two hydrophobic helices to span the membrane, with the A component proposed to provide amphipathic helices that produce the hydrophilic interior lining of the oligomeric pore (Wilson *et al.*, 2019 ▸; Mueller *et al.*, 2009 ▸; Benke *et al.*, 2015 ▸).

Tripartite α-PFTs were first identified in the pathogenic Gram-positive bacterium *Bacillus cereus*, when the Hbl system, and later the NheABC system, were identified as vital toxins in its pathogenicity and the cause of a major food-poisoning outbreak in Norway (Thompson *et al.*, 1984 ▸; Lund & Granum, 1996 ▸; Beecher *et al.*, 1995 ▸). The tripartite α-PFT family has recently been expanded into a large number of clinically and economically important Gram-negative bacteria, including the fish and opportunistic human pathogen *Aeromonas hydrophila* (Wilson *et al.*, 2019 ▸). The α-PFT toxin AhlABC from *A. hydrophila*, like NheABC, has been shown to be lytic to mammalian cells and forms pores in membranes (Wilson *et al.*, 2019 ▸; Lindbäck *et al.*, 2004 ▸).

Structures of soluble AhlB and AhlC, and also a pore structure of AhlB, have been solved by X-ray crystallography; however, a structure of the A component from a Gram-negative α-PFT has yet to be determined (Wilson *et al.*, 2019 ▸) and thus the structural role of this protein in the active pore is as yet unknown. Within the Gram-positive *B. cereus* α-PFTs, only the structures of NheA (PDB entry 4k1p; Ganash *et al.*, 2013 ▸) and HblB (PDB entry 2nrj; Madegowda *et al.*, 2008 ▸) have been determined. HblB is functionally equivalent to AhlC, yet these two proteins share less than 10% sequence identity and their structures are significantly different (Wilson *et al.*, 2019 ▸). Similarly, NheA and AhlA share only 6% sequence identity, and thus structures of the A component from the Gram-negative bacterial α-PFT systems may also vary substantially from that of NheA.

*Serratia marcescens* is a nosocomial human-pathogenic Gram-negative bacteria (Su *et al.*, 2003 ▸; Kurz *et al.*, 2003 ▸; Iguchi *et al.*, 2014 ▸). Genomic analysis has shown that it possesses an α-PFT with three proteins (SmhABC) homologous to the AhlABC proteins (Wilson *et al.*, 2019 ▸).

In this paper, we present the overexpression, purification and crystallization of SmhA and show the first electron-density map for an A component of a Gram-negative tripartite α-PFT.

## 2. Materials and methods   

### 2.1. Macromolecule production   

#### 2.1.1. Cloning and overexpression   

The open reading frame for SmhA from *S. marcescens* MSU97 (NCBI accession No. OKB64935.1) was synthesized and cloned into the pET-21a expression vector by GenScript to contain a C-terminal His_6_ tag.

The plasmid was transformed into an *Escherichia coli* BL21 (DE3) expression cell line (NEB). One colony was used to inoculate a 250 ml flask containing 50 ml Luria–Bertani (LB) broth supplemented with 100 µg ml^−1^ ampicillin and was grown overnight at 37°C. 10 ml of this overnight culture was then used to inoculate 500 ml LB broth supplemented and incubated as described above until an OD_600_ of 0.6 was reached, at which point protein expression was induced by the addition of 1 m*M* isopropyl β-d-1-thiogalactopyranoside (IPTG). Protein expression was carried out overnight at 16°C.

To prepare selenomethionine-incorporated SmhA, 2 × 500 ml of cells were grown as described above and harvested prior to induction. The cells were washed and resuspended in selenomethionine minimal medium [10.5 g l^−1^ K_2_HPO_4_, 1.0 g l^−1^ (NH_4_)_2_SO_4_, 4.5 g l^−1^ KH_2_PO_4_, 0.5 g l^−1^ trisodium citrate·2H_2_O, 5.0 g l^−1^ glycerol and 0.5 g l^−1^ each of adenine, guanosine, thymine and uracil; medium *A*] and added to 2 × 500 ml of medium *A* supplemented with 1.0 g l^−1^ MgSO_4_·7H_2_O, 4.0 mg l^−1^ thiamine; 100 mg l^−1^ each of l-lysine, l-phenylalanine and l-threonine; 50 mg l^−1^ each of l-isoleucine, l-leucine and l-valine; and 40 mg l^−1^ seleno-l-methionine. Growth was continued until an OD_600_ of 0.6 was reached before induction with 1 m*M* IPTG. The protein was expressed overnight at 16°C. The cells were harvested and pelleted before storage at −25°C.

#### 2.1.2. Purification   

Harvested cells of either native or selenomethionine-derivatized (SeMet) SmhA were defrosted, resuspended in lysis buffer (50 m*M* Tris pH 8.0) and lysed by sonication (3 × 20 s bursts at 16 µm amplitude). Insoluble material was removed by centrifugation at 40 000*g* for 15 min. The supernatant was applied onto a 5 ml nickel HiTrap column (GE Healthcare) in binding buffer (50 m*M* Tris pH 8.0, 0.5 *M* NaCl). The protein was eluted with a linear gradient of 0–1 *M* imidazole in binding buffer and fractions containing protein were pooled, concentrated and buffer-exchanged into 50 m*M* Tris pH 8.0, 10 m*M* NaCl for crystallization; the purification was analysed by SDS–PAGE. Macromolecule-production information is summarized in Table 1 ▸.

### 2.2. Crystallization   

Purified SmhA was concentrated to 7 mg ml^−1^ for crystallization using a Vivaspin 30 kDa molecular-weight cutoff concentrator (Sartorius). The concentrated protein was used to set up 96-well sitting-drop crystallization trials using a TTP LabTech Mosquito LCP robot, with both 200 nl:200 nl and 200 nl:100 nl well solution:protein solution drops, and stored at 7°C. Crystallization-condition suites used for preliminary screens included JCSG+, PACT *premier*, MPD, Morpheus, ProPlex and AmSO4 (Qiagen and Molecular Dimensions).

**Figure 1:**
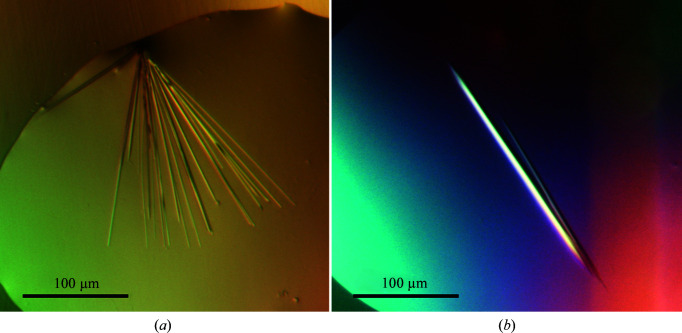
(*a*) Native SmhA crystals grown in PACT *premier* condition B11 (0.2 *M* MES pH 6, 0.2 *M* CaCl_2_, 20% PEG 6000). (*b*) SeMet SmhA crystals grown in optimized conditions based on PACT *premier* condition B11 (0.1 *M* MES pH 6.1, 0.14 *M* CaCl_2_, 21% PEG 6000).

Initial crystals of both native and SeMet SmhA grew in PACT *premier* condition B11 (0.2 *M* MES pH 6, 0.2 *M* CaCl_2_, 20% PEG 6000). Optimization (using a Formulatrix Formulator robot) of the SeMet SmhA crystals around PACT *premier* condition B11 in a 96-well sitting-drop plate with 200 nl:200 nl drops gave larger more defined crystals from 0.1 *M* MES pH 6.1, 0.14 *M* CaCl_2_, 21% PEG 6000 (Fig. 1 ▸). Crystallization information is summarized in Table 2 ▸.

### 2.3. Data collection and processing   

**Figure 2:**
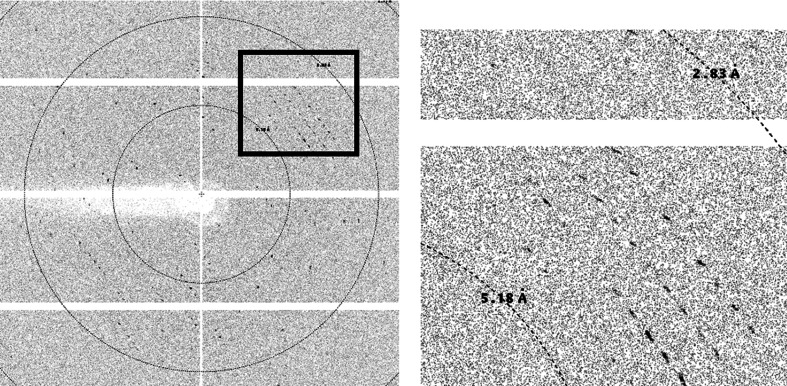
A representative 0.1° oscillation image from an SeMet SmhA crystal collected using an EIGER2 XE 16M detector on beamline I03 at Diamond Light Source. An enlarged view of the region highlighted by the square shows that diffraction extends to around 3.3 Å resolution.

A single SeMet SmhA crystal was flash-cooled in liquid nitrogen using a cryoprotectant consisting of 20% ethylene glycol, 0.2 *M* MES pH 6, 0.2 *M* CaCl_2_, 20% PEG 6000 and data were collected at the selenium absorption edge (0.9792 Å) on beamline I03 at Diamond Light Source (DLS; Fig. 2 ▸). Data were processed to 3.3 Å resolution using the *xia*2/*DIALS* pipeline (Winter *et al.*, 2018 ▸) and showed that the crystal belonged to space group *P*4_2_, with unit-cell parameters *a* = *b* = 151.8, *c* = 134.0 Å (Table 3 ▸).

## 3. Results and discussion   

### 3.1. Construct design   

**Figure 3:**
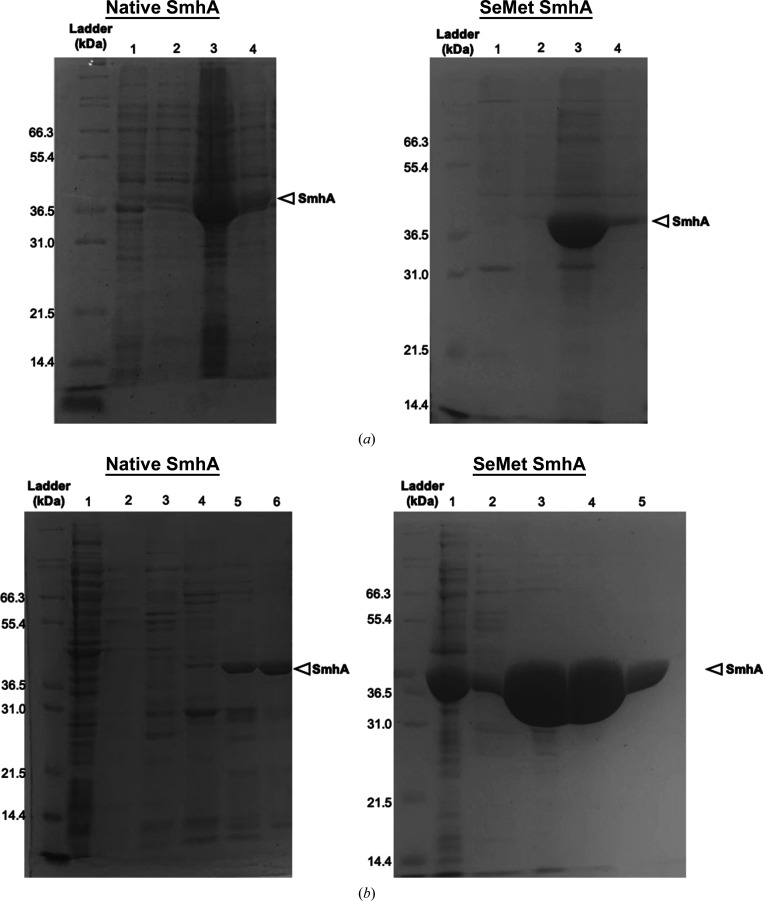
(*a*) SDS–PAGE gels showing overexpression of native and SeMet SmhA. Lane 1, pre-induction insoluble fraction; lane 2, pre-induction soluble fraction; lane 3, post-induction insoluble fraction; lane 4, post-induction soluble fraction. (*b*) SDS–PAGE gels showing nickel HiTrap column purification of native and SeMet SmhA. Lane 1, cell-free extract; lanes 2–5/6, elution fractions from the nickel HiTrap column. Fraction 6 and fractions 4 and 5 (native and SeMet, respectively) were >90% pure and were used for crystallization.

Structural studies of the tripartite toxins have been hampered in part by difficulties in producing large quantities of stable protein. The expression of *A. hydrophila* AhlA using constructs generated from genomic DNA in *E. coli* BL21 cells (Wilson *et al.*, 2019 ▸) produced protein for assays, but the yield was low and insufficient for crystallization. Pairwise sequence alignment of AhlA with *S. marcescens* SmhA shows 43% identity and 53% similarity (Supplementary Fig. S1), identifying SmhA as a good candidate for structural studies of the A component from a Gram-negative bacterium. As with AlhA, initial attempts to overexpress SmhA using constructs from genomic DNA also proved unsuccessful. To try to improve expression in *E. coli* BL21, the SmhA gene (NCBI accession No. OKB64935.1) was synthesized and optimized in both GC content and codon usage for expression in *E. coli* (GenScript). This resulted in 38% of the codons being altered (Supplementary Fig. S2). This new construct was successfully used to express SmhA in *E. coli* with a C-terminal 6×His tag and, following nickel column purification, resulted in protein with >90% purity and a good yield (9.5 mg l^−1^; Fig. 3 ▸).

### 3.2. Data analysis of SmhA   

**Figure 4:**
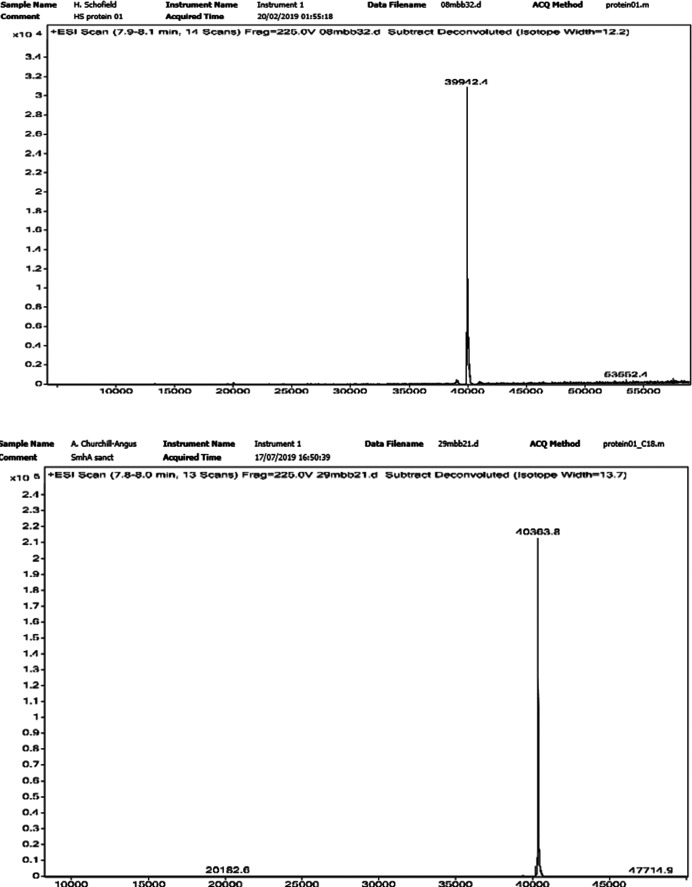
Mass spectrum for native SmhA (top) and SeMet SmhA (bottom) as used for crystallization. A molecular weight of 40 363.8 Da for SeMet SmhA (the molecular weight of native SmhA with a His_6_ tag is 39 942.4 Da) shows the incorporation of selenium at all nine methionine sites.

**Figure 5:**
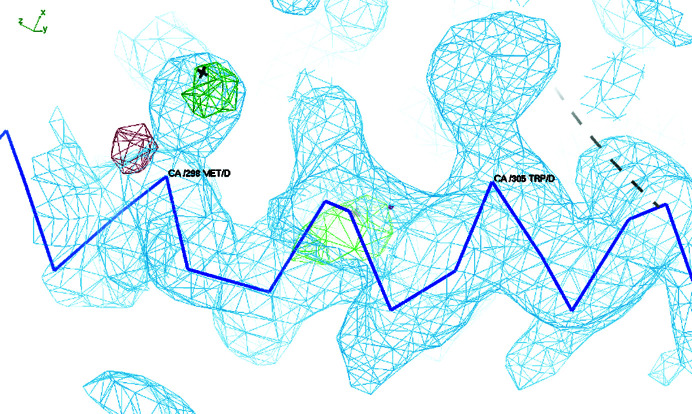
Initial electron-density map contoured at 1.0σ (blue) and an anomalous difference map (positive, green) showing a helical section of SmhA. Density can be seen for the side chains of Trp305 and Met298, with positive difference for the Se atom (shown as a cross) in Met298.

Analysis of the Matthews coefficient for SeMet SmhA showed that the asymmetric unit most likely contained between six and ten molecules with a solvent content between 63% and 38%, respectively, with eight molecules being the most probable, with a *V*_M_ value of 2.39 Å^3^ Da^−1^ and a solvent content of 48% (Matthews, 1968 ▸; Kantardjieff & Rupp, 2003 ▸). Mass spectrometry showed that the molecular weight of SeMet SmhA was 40 363.8 Da, which is 421.4 Da more than the native sample, indicating full incorporation of Se atoms into the nine methionine residues of the protein and also indicating that the protein was of high purity (Fig. 4 ▸). In order to maximize the quality of the single-wavelength selenium anomalous signal, data-collection parameters were chosen to minimize radiation damage, whilst still providing good multiplicity, albeit at the expense of resolution. A beam size of 80 × 20 µm was selected to match the dimensions of the crystal, with a beam transmission of 20%, giving a flux of 7.44 × 10^11^ photons s^−1^. 3600 images of 0.1° and an exposure of 0.008 s gave a data set with a half-set correlation coefficient of 1.0, an anomalous multiplicity of 7.0 and an anomalous correlation coefficient of 0.2 with no obvious signs of radiation damage. Selenium positions were calculated from these Se SAD data, and an initial density map and model were generated using the *CRANK*2 pipeline (Skubák & Pannu, 2013 ▸; Fig. 5 ▸). A preliminary initial model of SmhA, placing 2809 residues assigned to 28 fragments, was automatically built into the electron density. Visual inspection of the map and model confirmed that eight molecules were present in the asymmetric unit, with the side-chain positions of the methionine residues clearly aligned with the positive density of the anomalous difference map. A self-rotation function calculated using the data between 50 and 6 Å resolution showed the presence of a noncrystallographic twofold axis perpendicular to the crystallographic fourfold (peak of 88% of the origin at polar coordinates 90.0, 111.2, 180°). Inspection of the initial model showed that two subunits were related by this rotation axis, but were separated by 20 Å. In addition, a self-Patterson indicated the presence of noncrystallographic translational symmetry with a peak of 43% of the origin at 0, 0, 0.467 and the model showed that six of the eight molecules were related by this translational symmetry. However, despite these non­crystallographic symmetry relationships, no high-order oligomeric arrangement could be observed for the eight subunits, indicating that the structure of SmhA was of the monomeric soluble form of the protein, rather than an oligomeric structure assembled around a central rotation axis as required for the proposed pore form.

Further work to extend the resolution of the data and refine the SmhA structure is ongoing.

## Supplementary Material

Supplementary figures. DOI: 10.1107/S2053230X20013862/ow5025sup1.pdf

## Figures and Tables

**Table 1:** Macromolecule-production information

Source organism	*S. marcescens* MSU97
Restriction sites	NdeI/XhoI
Cloning vector	pET-21a(+)
Expression vector	pET-21a(+)
Expression host	*E. coli* BL21 (DE3)
Complete amino-acid sequence of the construct produced	MNNLTSIDLSPQTLMAMHISISSQALLNQSYSNLLLSQQLLTSQSMDPGLTVKIKAYQNQLRQQAQVFKQNTVAELIGLYTKASNFAALVNAVNALYSTEDPQVSQKGAEMVAALSDVAQHYQAAAQAVHTQLQAKREMLEPLMGNFLNVIDAIEQGLNAEAKQQAQTIAELNEAIAKNIQSIADAGFKAGEGVVQLGQSIVAAVPLGPTDKKPKEAPTAPPKPLSDQASYMISGIQAISAGASGAQQAVNELKANYAKLAVAYRALATANALLSVAKSVQAQAQLFVDTYVLTEQRMALLPTEWGKVAEAYLTAAPIINQAGSAAEIKQAKQIISLNAEKWQLFSKSIDNAKANYAGNNILPEVLEHHHHHH

**Table 2:** Crystallization

Method	Sitting-drop
Plate type	96-well sitting drop
Temperature (K)	280
Protein concentration (mg ml^−1^)	7
Buffer composition of protein solution	50 m*M* Tris pH 8.0, 10 m*M* NaCl
Composition of reservoir solution	0.2 *M* MES pH 6, 0.2 *M* CaCl_2_, 20% PEG 6000
Volume and ratio of drop	200 nl:200 nl
Volume of reservoir (µl)	50

**Table 3:** Data collection and processing Values in parentheses are for the outer shell.

Diffraction source	I03, DLS
Wavelength (Å)	0.9792
Temperature (K)	100
Detector	EIGER2 XE 16M
Rotation range per image (°)	0.1
Total rotation range (°)	360
Exposure time per image (s)	0.008
Space group	*P*4_2_
*a*, *b*, *c* (Å)	151.8, 151.8, 134.0
α, β, γ (°)	90, 90, 90
Mosaic spread (°)	0.19
Resolution range (Å)	67.9–3.34 (3.40–3.34)
Total No. of reflections	612546 (30358)
No. of unique reflections	44270 (2175)
Completeness (%)	100 (98.5)
Multiplicity	13.8 (14.0)
〈*I*/σ(*I*)〉†	4.7 (1.0)
*R*_r.i.m._	0.118 (0.759)
Overall *B* factor from Wilson plot (Å^2^)	56.626

† The high-resolution cutoff for the data was automatically determined in the *DIALS* pipeline, which uses CC_1/2_ = 0.5 as the limit of usable data. For the outer shell the mean *I*/σ(*I*) is 2.0 at 3.7 Å resolution.
