# Supplementary material for: The A component (SmhA) of a tripartite pore-forming toxin from Serratia marcescens: expression, purification and crystallographic analysis
Source: Acta Crystallogr F Struct Biol Commun. 2020 Nov 25;76(Pt 12):577–82. doi: 10.1107/S2053230X20013862 (PMC7716259; doi:10.1107/S2053230X20013862)
Supplement: Supplementary file 1 [file f-76-00577-sup1.pdf]

STRUCTURAL BIOLOGY
COMMUNICATIONS

Volume 76 (2020)

Supporting information for article:

The A component (SmhA) of a tripartite pore-forming toxin from *Serratia marcescens*: expression, purification and crystallographic analysis

Alicia M. Churchill-Angus, Svetlana E. Sedelnikova, Thomas H. B. Schofield and Patrick J. Baker

```
         1        10        20        30        40        50        60
AhlA  MTTIATLDLGDQGLLAAYLSATSPALLNQTSANLLLMQQSLANSEIDSTLAVKIEAYQAQ
SmhA  MNNLTSIDLSPQTLMAMHISISSQALLNQSYSNLLLSQQLLTSQSMDPGLTVKIKAYQNQ

                  70        80        90       100       110       120
AhlA  MMNQARYYQQTNLSGLIHLLSNGSNFYALVAAFNRLLGQEDDAAVLAGGARLGQQLTSLV
SmhA  LRQQAQVFKQNTVAELIGLYTKASNFAALVNAVNALYSTEDPQVS.QKGAE...MVAALS

                 130       140       150       160       170       180
AhlA  DQAQSYRLNAQALNTLMQVAWSSLRPLMTQFNGVISQLEQDLMADVKRQTEIIDGLNKAI
SmhA  DVAQHYQAAAQAVHTQLQAKREMLEPLMGNFLNVIDAIEQGLNAEAKQQAQTIAELNEAI

                 190       200       210       220       230       240
AhlA  AANIQAIVDEGGKAGEGVNQLGRAIVTSLTLEQKPADKGAKPATSQEIAKETDQQTQYMI
SmhA  AKNIQSIADAGFKAGEGVVQLGQSIVAAVPL..GPTDKKPKEAPTAPP.KPLSDQASYMI

                 250       260       270       280       290       300
AhlA  SGLEALSSGIAGSSQAARELKTNTDKLAQAYQALAATNALLSVAKSVQAQNQLFVDTYSR
SmhA  SGIQAISAGASGAQQAVNELKANYAKLAVAYRALATANALLSVAKSVQAQAQLFVDTYVL

                 310       320       330       340       350       360
AhlA  VADTVTQLPAGWQKVADAYQGAVPVVTDLNSPDDVRQLRRTVALNTQSWQLLSSQVDDIK
SmhA  TEQRMALLPTEWGKVAEAYLTAAPIINQAGSAAEIKQAKQIISLNAEKWQLFSKSIDNAK

                 370
AhlA  AAYAGNGILPDA..
SmhA  ANYAGNNILPEVLE
```

Supplementary figure S1. Pairwise sequence alignment of AhlA and SmhA. Red boxes with white text shows identical residues, while white boxes with red text shows similar residues.

```
Optimized      4   ATGAACAACCTGACCAGCATCGACCTGAGCCCGCAAACCCTGATGGCGATGCACATCAGC
Original       4   ATGAATAACTTGACGAGTATTGATTTAAGCCCGCAAACACTAATGGCAATGCATATTTCC

Optimized     64   ATTAGCAGCCAAGCGCTGCTGAACCAGAGCTACAGCAACCTGCTGCTGAGCCAGCAACTG
Original      64   ATTTCATCACAGGCATTGCTTAATCAAAGCTATAGTAATTTGTTGCTCAGCCAGCAATTA

Optimized    124   CTGACCAGCCAGAGCATGGACCCGGGTCTGACCGTGAAGATTAAAGCGTATCAGAACCAA
Original     124   TTAACGTCACAAAGTATGGACCCTGGCCTGACCGTTAAAATCAAGGCGTACCAAAATCAA

Optimized    184   CTGCGTCAGCAAGCGCAAGTGTTCAAACAGAACACCGTTGCGGAGCTGATCGGCCTGTAC
Original     184   CTGCGGCAGCAGGCGCAAGTATTCAAACAAAATACGGTCGCAGAATTGATCGGGTTATAT

Optimized    244   ACCAAGGCGAGCAACTTTGCGGCGCTGGTGAACGCGGTTAACGCGCTGTATAGCACCGAG
Original     244   ACCAAAGCGTCAAATTTCGCCGCCCTGGTCAACGCCGTCAATGCGCTGTATTCCACCGAG

Optimized    304   GACCCGCAAGTTAGCCAGAAGGGTGCGGAAATGGTGGCGGCGCTGAGCGATGTTGCGCAA
Original     304   GATCCTCAGGTGTCGCAGAAAGGCGCAGAAATGGTGGCTGCGCTTTCCGATGTCGCGCAA

Optimized    364   CACTATCAAGCGGCGGCGCAGGCGGTGCACACCCAGCTGCAAGCGAAACGTGAGATGCTG
Original     364   CACTACCAGGCCGCAGCACAAGCCGTCCACACGCAGTTGCAGGCGAAACGCGAAATGCTG

Optimized    424   GAACCGCTGATGGGTAACTTCCTGAACGTTATCGACGCGATTGAGCAAGGCCTGAACGCG
Original     424   GAGCCTTTGATGGGCAATTTCCTTAACGTCATTGACGCCATCGAACAAGGGCTGAACGCC

Optimized    484   GAAGCGAAGCAGCAAGCGCAGACCATTGCGGAGCTGAACGAAGCGATCGCGAAAAACATC
Original     484   GAGGCGAAACAACAGGCGCAAACCATCGCTGAGCTCAACGAAGCGATCGCCAAAAATATC

Optimized    544   CAGAGCATTGCGGATGCGGGTTTTAAGGCGGGCGAGGGCGTGGTTCAACTGGGTCAGAGC
Original     544   CAAAGCATCGCAGATGCGGGTTTCAAAGCGGGTGAAGGGGTTGTGCAGCTTGGACAATCC

Optimized    604   ATTGTTGCGGCGGTTCCGCTGGGTCCGACCGACAAGAAACCGAAAGAAGCGCCGACCGCT
Original     604   ATCGTCGCCGCCGTGCCGCTCGGCCCGACGGATAAAAAACCGAAAGAAGCGCCAACCGCG

Optimized    664   CCGCCGAAGCCGCTGAGCGATCAAGCGAGCTATATGATCAGCGGCATTCAGGCGATCAGC
Original     664   CCTCCGAAGCCGCTTAGCGACCAGGCCAGCTATATGATCTCCGGCATTCAGGCGATCTCG

Optimized    724   GCGGGTGCGAGCGGTGCGCAGCAAGCGGTGAACGAGCTGAAGGCGAACTACGCGAAACTG
Original     724   GCGGGCGCTTCCGGTGCACAACAGGCCGTTAATGAACTGAAGGCAAACTACGCCAAATTG
```

```
Optimized    724    GCGGGTGCGAGCGGTGCGCAGCAAGCGGTGAACGAGCTGAAGGCGAACTACGCGAAACTG
Original     724    GCGGGCGCTTCCGGTGCACAACAGGCCGTTAATGAACTGAAGGCAAACTACGCCAAATTG

Optimized    784    GCGGTTGCGTACCGTGCGCTGGCGACCGCGAACGCGCTGCTGAGCGTGGCGAAAAGCGTT
Original     784    GCCGTGGCCTATCGGGCTTTGGCAACCGCCAATGCGCTGCTCTCCGTCGCCAAGTCCGTG

Optimized    844    CAGGCGCAAGCGCAGCTGTTCGTGGACACCTACGTTCTGACCGAACAACGTATGGCGCTG
Original     844    CAAGCCCAGGCCCAATTATTTGTCGACACCTATGTTCTCACCGAGCAGCGCATGGCGTTA

Optimized    904    CTGCCGACCGAGTGGGGTAAAGTGGCGGAAGCGTATCTGACCGCGGCGCCGATCATTAAC
Original     904    CTGCCGACAGAATGGGGCAAGGTGGCGGAAGCCTATCTGACGGCTGCGCCCATCATTAAC

Optimized    964    CAAGCGGGCAGCGCGGCGGAGATCAAGCAAGCGAAACAGATCATTAGCCTGAACGCGGAA
Original     964    CAAGCCGGCAGCGCAGCGGAAATTAAACAAGCCAAACAAATCATTTCGCTCAATGCGGAA

Optimized   1024    AAGTGGCAGCTGTTTAGCAAAAGCATTGATAACGCGAAGGCGAACTACGCGGGCAACAAC
Original    1024    AAATGGCAGCTGTTTTCAAAGTCTATTGATAACGCCAAAGCTAACTATGCAGGAAACAAC

Optimized   1084    ATCCTGCCGGAAGTT
Original    1084    ATCCTGCCGGAAGTT
```

Supplementary figure S2. Optimized and unoptimized nucleotide sequence for SmhA. Altered nucleotides are highlighted in red.
